# Mosquito direct skin feeding bioassay: 15 years of experience and a standardised approach in Mali

**DOI:** 10.1186/s12936-025-05726-7

**Published:** 2026-02-04

**Authors:** Daman Sylla, Jen C. C. Hume, Heather Goodman, Adama Sacko, Jennifer L. Kwan, Abdrahamane Fofana, Mahamadoun H. Assadou Maiga, Abdoulaye Katile, M.’bouye Doucoure, Mamady Kone, Boubacar Coulibaly, Agnes Guindo, Moussa Diallo, Mariam Doumbia, Salifou M. Kone, Amatigue Zeguime, Sekou Goita, Moridie Sidibe, Sale Sidibe, Yacouba Dembele, Kourane Sissoko, Baba Djiguiba, Seydou Sankare, Sadio D. K. Diarra, Ousmane A. Poudiougo, Bakary Traore, Yacouba Diarra, Lakamy Sylla, Boubacar Tembely, Amadou Sekou Traore, Bourama Kamate, Ibrahima Baber, Alemush Imeru, Emily Higbee, Olga Muratova, Freda Omaswa, Yimin Wu, Alpha S. Yaro, Sara A. Healy, Issaka Sagara, Cheick S. Traore, Ogobara K. Doumbo, Mamadou B. Coulibaly, Patrick E. Duffy

**Affiliations:** 1 Malaria Research and Training Center, University of Sciences, Techniques and Technologies of Bamako, Bamako, Mali; 2 Laboratory of Malaria Immunology and Vaccinology, National Institute of Allergy and Infectious Diseases, National Institutes of Health, Bethesda, MD USA; 3 Laboratory of Clinical Immunology and Microbiology, National Institute of Allergy and Infectious Diseases, National Institutes of Health, Bethesda, MD USA

**Keywords:** Malaria, Transmission, Direct skin feeding bioassay, Mosquitoes, Transmission-blocking vaccines

## Abstract

**Background:**

New malaria control tools are urgently needed. Transmission-blocking vaccines (TBVs) target sexual parasite stages in mosquitoes to prevent disease spread. TBV testing requires specialised mosquito transmission assays, such as the Direct Skin Feeding (DSF) bioassay. DSF is a xenodiagnostic tool that mimics parasite transmission to mosquitoes as it naturally occurs but has not previously been scaled up nor standardised for use in clinical trials.

**Methods:**

DSF bioassays were performed on large cohorts of participants, including children five years and older, during observational and interventional studies conducted over a 15-year period at sites around Bamako, Mali. Human, mosquito and parasite parameters were monitored to assess DSF safety and acceptability, vector performance, and individual- and population-level transmission dynamics. Standardised procedures developed for DSF included mosquito colony maintenance, mosquito preparation for DSF, feeding procedures, quality control metrics, ethical approaches and volunteer follow-up.

**Results:**

From 2011 through 2025, 37,984 DSF bioassays were performed on 2,796 rural study participants aged 5 years and older, at two Mali study sites. DSFs were well-accepted and safe, with a low refusal rate (0.8% of subjects in vaccine studies) and rare adverse events (AE) that met grading criteria (11 subjects; 0.032%). The few immediate and short-term skin reactions that met AE grading criteria were mild or moderate in severity, all resolving within 48 h. DSF infrastructure was progressively scaled up to a capacity of 120 assays per day requiring 36,000 female mosquitoes per week. Rates of DSF positivity were highest in studies where feeds were conducted on individuals with known *Plasmodium falciparum* parasitaemia (average 18.4%) vs studies where feeds were conducted on all participants irrespective of blood smear status (average 1.6%).

**Conclusions:**

The DSF bioassay is a xenodiagnostic tool to detect transmissible malaria parasites, and a scalable and safe method for evaluating TBV efficacy. DSF offers several advantages including close mimicry of naturally occurring transmission, simplicity of performance and standardisation, and scalable throughput to support late-stage vaccine trials. While parasite transmission rates measured by DSF were low overall at study sites in Mali, sufficient transmission endpoints are generated to assess efficacy of interventions that interrupt transmission, supporting the DSF bioassay as a surrogate efficacy endpoint for TBV studies.

**Supplementary Information:**

The online version contains supplementary material available at 10.1186/s12936-025-05726-7.

## Background

Progress in global malaria control has plateaued, and reversed in some high transmission regions, lending urgency to the search for new control tools. RTS,S and R21 malaria vaccines have been licensed and are being implemented through public health systems in many sub-Saharan countries, with the indication to protect young children from clinical malaria and death [1, 2]. Both vaccines target the pre-erythrocytic stage of *Plasmodium falciparum*, preventing invasion of liver cells and progression to disease-causing blood stages. While these vaccines represent a historic achievement in malaria control, neither is indicated for older children and adults, thus additional measures remain necessary to pursue elimination.

Transmission Blocking Vaccines (TBV) that prevent or reduce human-to-mosquito transmission offer a new class of interventions and have been endorsed by the World Health Organization (WHO) as a potential strategy [3]. TBVs target sexual stage parasites within the mosquito, preventing infection of new human hosts and spread of disease within a community, thereby offering herd immunity protection to vaccinees and non-vaccinees alike. TBVs can be combined with anti-infection vaccines as multistage products to prevent infection of both the mosquito and human hosts, targeting key bottlenecks in parasite development and providing direct benefits to vaccinees.

TBVs require specialised mosquito transmission assays to measure their activity or efficacy. Three mosquito transmission assays have been used: Standard Membrane Feeding Assay (SMFA), Direct Membrane Feeding Assay (DMFA), and in the context of TBV field trials, Direct Skin Feeding (DSF) bioassay. For decades, the SMFA has been considered a gold standard assay for evaluating activity of TBV candidates such as Pfs25 [4, 5] and Pfs230D1 [6–8]. However, SMFA is a low throughput assay that requires specialised laboratory infrastructure and relies on laboratory-reared mosquitoes and parasite strains, making it less representative of natural transmission; SMFA assay endpoints are influenced by intensity of mosquito infections which can vary widely between experiments. In contrast, both DSF and DMFA are deployed in field settings and measure transmission of naturally circulating parasite strains, either by feeding naive mosquitoes directly on volunteers (DSF) or across a membrane (e.g., Parafilm® or Baudruche) on infected blood held in an artificial feeding device (DMFA). Both assays measure transmission dynamics in a natural setting and have improved the understanding of transmission of vector-borne diseases [9].

DSF is a xenodiagnostic assay that relies on pathogen transmission to the mosquito vector and has been used in diverse settings for different mosquito-borne diseases such as malaria [10] and dengue [11]. The earliest descriptions of malaria transmission by Ronald Ross and Giovanni Grassi utilised DSF to infect mosquitoes [12, 13]. Mosquito feeding experiments have revealed infectiousness of gametocyte carriers [14, 15] and susceptibility of different mosquito species to malaria parasites [16–19]. Thus, DSF is a long-established tool with biological plausibility for measuring malaria parasite transmission and by extrapolation may be useful for measuring efficacy of TBV or other transmission-blocking interventions [4, 8].

While the different mosquito feeding assays are widely used, the scale up and standardisation of DSF for use as a surrogate measure of TBV efficacy has been pioneered in Mali over recent years [4, 8, 15]. As TBV candidates developed at the US National Institutes of Health advanced to field trials, studies optimised and compared DMFA and DSF when performed on Malian adults and children [9, 20]. Optimisation parameters included mosquito age at time of feeding, mosquito starvation time, mosquito feeding location, time of day for feeding, and for DMFA only, the membrane material for the feeding apparatus. As noted in a 2016 systematic review [21], DSF is a critical tool to assess transmission-blocking interventions but has lacked data to demonstrate scalability and standardisation.

For both DSF and DMFA, the low frequency of transmission events at a population level, even during peak malaria season, has yielded insufficient endpoints to assess TBV efficacy. Recent published data from mosquito feeding experiments involved a relatively small number of volunteers [11] who each participated in only one or a few feeding sessions [22], which is insufficient when measuring vaccine efficacy.

This manuscript describes the experience of a single research centre operating at two field sites over 15 years to scale up, optimise and standardise the DSF bioassay, including measures of quality control and assay reproducibility. With data from over 37,000 assays, this manuscript reports the largest body of data compiled on direct mosquito feeding as a study endpoint. DSF safety and tolerability across different age groups including children, quality control procedures for mosquito rearing, standardisation of the DSF bioassay procedure, and guidance on interpreting and analysing data as a TBV surrogate efficacy endpoint are described.

## Methods

### Study site description

These studies were conducted as a collaboration between the Laboratory of Malaria Immunology and Vaccinology, National Institute for Allergy and Infectious Diseases, National Institutes of Health (LMIV/NIAID/NIH) in the US and the Malaria Research and Training Center, University of Sciences, Techniques, and Technologies-Bamako (MRTC/USTTB) in Mali. Field studies were conducted at two rural locations in Mali, West Africa – Bancoumana and Donéguébougou, along with surrounding villages.

Bancoumana is located 60 kms southwest of Bamako and currently has a population of about 23,000 people. The site is situated in the south-Sudanian area of Mali. The climate is hot, with daily temperatures ranging from 19 °C to 40 °C. The annual rainfall varies between 600 and 1200 mm with a main rainy season occurring from June to October with a resulting malaria season typically running from July through December. Many clinical trials, as well as epidemiological and entomologic malaria studies, have been conducted in Bancoumana.

Donéguébougou is a community located 30 kms north of Bamako and has a population of about 2,000, with another 2,000 inhabitants in the surrounding villages. Like Bancoumana, malaria transmission is highly seasonal and usually occurs from July through December. Donéguébougou is situated in a high transmission area, with entomological inoculation rates (determined by human landing catch) as high as 137 to 167 infectious bites per person over one transmission season [23].

### Study details

Data analysed for this report were drawn from 8 separate protocols (comprising 9 studies) conducted between 2011–2025 including four observational cohort studies, one stand-alone feeding protocol for whole organism vaccine trial subjects, and four TBV clinical trials. Data included in this manuscript include DSF conducted on all study participants (vaccinees and comparators where relevant), except when reporting mosquito infectivity results where only data from observational studies and from comparator vaccine recipients in vaccine trials are included. Each study had specific eligibility criteria leading to variation in age of study participants, and studies also varied in the number and frequency of DSF bioassays performed (study details in Table 1**)**.

**Table 1:** Summary of studies that incorporated DSF bioassay

Study Name	Clinicaltrials.gov Registration Number	Year (s) Conducted	Study Site	DSF Age Range per Protocol	N DSF Performed	N DSF Participants
Observational Transmission study	NCT01360112	2011–2015	Bancoumana	5–50 years	178	105
Pfs25-EPA/Alhydrogel	NCT01867463	2013–2015	Bancoumana	18–45 years	630	93
Pfs25-EPA/Alhydrogel and Pfs230D1-EPA/Alhydrogel	NCT02334462	2015–2016	Bancoumana	18–50 years	3850	175
Standalone Mosquito feeding study	NCT02206451	2014–2015	Doneguebougou	18–35 years	500	87
Pfs230D1/AS01 in adults	NCT02942277	2017–2018	Bancoumana and Doneguebougou	18–50 years	9931	217
Community transmission study	NCT03304704	2018–2020	Bancoumana and Doneguebougou	5–65 years	13,231	1217
Pfs230D1-AS01 Community Trial	NCT03917654	2019–2021	Doneguebougou	9–18 years	5944	380
DSF Study 1	2023/250/CE/USTTB*	2023–2024	Bancoumana	5–18 years	252	122
DSF Study 2	2023/250/CE/USTTB*	2024–2025	Bancoumana	5–18 years	3468	400

*Asterisks indicate studies that were not registered on clinicaltrials.gov

**Ethical considerations.**

All study protocols were submitted and approved by the Faculty of Medicine-Odonto-Stomatology (FMPOS) (2011–43, 2013–38, 2014/79, 2015/16/CE/FMPOS, 2016/133/CE/FMPOS, 2017/182/CE/FMPOS, 2019/10/CE/FMPOS, 2023/250/CE/USTTB) and all but one observational study (2023/250/CE/USTTB) by the National Institute of Health (NIH) or the National Institute of Allergy and Infectious Disease (NIAID) IRB (11-I-N143, 13-I-N109, 14-I-N159, 15-I-0044, 17-I-N006, 17-I-N180, 19-I-N086). All trials followed International Council for Harmonisation Good Clinical Practice guidelines and Malian regulations; community permission was obtained from village chiefs and elders and then individual written informed consent from all participants. All trials and studies except 2023/250/CE/USTTB were registered on clinicaltrials.gov (Table 1).

### Mosquito colony maintenance and quality control

DSF in Mali were performed with female *Anopheles coluzzii*, previously identified as molecular form M of *Anopheles gambiae,* but designated a distinct species in 2013 [24]. The colony originated from collections conducted in N’gabakoro, Mali in 2008. Mosquitoes were genetically typed by the Entomology department of MRTC to be *An. coluzzii*. Quality assurance requirements are documented for personnel, facilities, reagents, and mosquitoes (Table 2).

**Table 2:** DSF quality assurance requirements

	Requirement
Personnel	4 main teams: mosquito production team, dissection team, midgut reading team, and a field team for performing the assays. Training programme for all activities; extended training for certified midgut readers
Facilities	Arthropod containment level (ACL)- compliant insectary compartmented into a rearing area, a secure area for holding infected mosquitoes post DSF, and a dissection and reading area for post- DSF analyses
Equipment	Rearing room: Modified cold room box which is temperature- regulated via air conditioners and humidifiers. Area is equipped with shelves, trays for larvae, cages for adult mosquitoes, a sink, an artificial feeding system (warm circulating bath connected to glass feeders) for feeding female mosquitoes for colony egg production Secure room: Wire mesh walls and door prevent escapees and allow adequate airflow for temperature and humidity maintenance. This area is equipped with shelves on which purpose-built wooden cases designed to hold individual DSF cups can be securely stored. Each wooden case holds 70 DSF cups Dissection/reading room: Ambient temperature room outfitted with multiple dissection and light microscopes. Dissection and reading space are separated via mesh walls and door with dissection space connected to secure room via pass-through window for cup transfer
Reagents	Consistent vendors; adequate labelling and storage
Process Performance	Assessed by mosquito feeding rate; mosquito survival rates from feed to dissection; mosquito infection prevalence and intensity. Data generated reviewed and QC'd internally and externally
Documentation	Full traceability of activities through electronic case report forms and paper source documents. Positive assay outcomes validated by photograph and post hoc molecular analyses
SOPs	Written SOPs for all activities with training verification
Archives	Source documents archived per sponsor requirements; stored specimens (infected midguts) stored and archived per study procedures
Audits	External independent audits

QC: Quality control; SOP Standard Operating Procedure

Mosquitoes were reared under standard insectary conditions (temperature 26 ± 2℃ and 75–85% relative humidity), with a photoperiodicity of 12 h light/dark. The colony was maintained on transfusion-grade O + human blood from the Centre National de Transfusion Sanguine in Bamako – blood was screened for HIV, hepatitis B, hepatitis C and syphilis per Mali blood transfusion guidelines. Blood donors were interviewed a week following donation to confirm no intercurrent febrile illnesses before blood was used for mosquito feeding. Naïve, female mosquitoes were fed on blood through an artificial membrane feeding system using Parafilm® on two consecutive days for up to 30 min and allowed to oviposit two days later. Eggs hatched in the egg container before being transferred to larval trays.

Two-day old larvae were placed into plastic trays at a density of approximately 200 larvae per litre of deionized water and fed with powdered TetraPond Koi Vibrancefish food (Koi; UK). Larvae were maintained in trays through pupation when pupae were collected using a netted spoon and placed into cages for emergence. Adult mosquitoes were fed on 10% sugar solution.

Rearing and maintenance procedures described above were followed regardless of the scale of production to ensure consistency in mosquitoes. To date, the Mali infrastructure has enabled up to 600 DSF to be conducted per week, involving 36,000 female mosquitoes. At this scale, mosquito production comprises multiple rolling batches of eggs being reared on a weekly basis, with a daily operation of selecting pupae and sorting adults for assays. Production was calculated to allow 5% overage on females for assays, and 30% additional production of females for colony maintenance.

To ensure quality control and consistency, mosquito batches were monitored continuously for parameters such as egg batch size, time to pupation, adult emergence rate and mosquito survival. Any mosquito batches that showed substantial deviations were not used for either feeding assays or colony maintenance. If deviations persisted, the colony underwent a performance review where multiple parameters including male and female longevity, wing length, time to pupation and sex ratio of emerged adults were measured in multiple replicates and compared to colony standards (based on averages from previously collected data). If any measures fell outside the MRTC SOP defined ranges for *An. coluzzii*, then corrective actions were taken, such as additional temperature/humidity monitoring of rooms, confirmation of larval rearing density, or confirmation of larval feeding schedule. In addition to mosquito development performance measures, the colony was also monitored routinely by PCR for Rift Valley Fever Virus (RVFV) infection by post-hoc testing of all blood units used for colony maintenance, as well as surveillance of adult colony mosquitoes (males and females). Although RVFV testing is not required for transfusion-grade blood per Malian national policy, the practice of post hoc screening was implemented per CDC recommendation, who cited limited reports that *Anopheles* mosquitoes might transmit RVFV transovarially [25]. Mosquitoes used in DSF assays have not previously fed on blood, and all blood units used to feed mosquitoes for colony maintenance are screened for RVFV.

### Mosquito preparation for DSF and standardisation

Newly emerged female adult mosquitoes aged 3–5 days old were sorted into cups for feeding assays. Cups used were modified 8-oz coffee cups (diameter 3 inches) in which the top was covered with fine tulle mesh secured with an elastic band and tape. A small hole was drilled in the bottom of the cups. Mosquitoes were moved into the feeding cups through the hole using mouth aspiration with trained operators counting the number of mosquitoes added until the desired number of mosquitoes per cup was obtained (i.e. 30 females as standard). Once the cup was filled, the hole in the base of the cup was plugged securely using dry cotton wool.

Prepped DSF cups were maintained on sugar solution and, if needed, wet cotton pads were added to maintain humidity. Eight hours prior to DSF bioassay, mosquitoes were moved from sugar solution to water only, and this was removed 2–4 h prior to scheduled feeding time. Thus, mosquitoes were starved for a total of 10–12 h and cups were starved in batches so that appropriately starved cups of mosquitoes were available to accommodate the sequential arrival of study participants for feeding assays conducted throughout the day.

Mosquitoes in feeding cups were transported from the main rearing insectary to field sites via a secondary cooler with an internal temperature tracker. Care was taken to avoid unnecessary bumping and jostling in transit, and the tracker was monitored upon arrival to ensure mosquitoes did not overheat. At the field site, mosquitoes were maintained in a ‘field insectary’ – a modified room designed to maintain standard conditions of the main insectary. Water pads over the cages helped maintain humidity when needed. Ideally, mosquitoes were allowed to settle for several hours or overnight in the field insectary before commencing feeding activities, and to rest overnight post-feeding before returning to the main insectary.

### DSF eligibility and standardisation

Eligibility criteria varied between protocols; for example, a number of the interventional trials only enrolled adult volunteers (Table 1). For all studies, participants completed a DSF eligibility questionnaire prior to each feeding assay, which determined any history of reaction to mosquito bites or anaphylaxis. Staff also recorded medical history and temperature before each DSF, and assays have only been performed on healthy individuals (which excludes those who are unwell with malaria or other illness, or recording a temperature of > 37.5 °C). Eligibility was documented on study-specific case report forms (CRFs) and only individuals deemed eligible were moved from the clinical rooms to the entomology feeding room, accompanied by a hard-copy patient liaison form used to track visit activities. A Participant Biometric Matching System (PBMS) was recently introduced to identify and monitor study participants during their clinic visits, establishing a record for each study participant during their screening visit after signing the informed consent form and agreeing to participate in the study. The system used facial recognition to identify participants during future visits, who received a printed wrist band upon check-in for each subsequent clinic visit that was returned at departure. When participants arrived for DSF, the entomology team confirmed the participant ID on their wristband against their liaison sheet and opened the matching electronic CRF to confirm eligibility.

For studies where DSF was only performed on individuals with a positive malaria blood smear, study participants would come in on day 1 for blood smear and those that met protocol defined infection criteria (presence of parasites and/or gametocytes only on blood smear) would get called to return on day 2. On day 2, the individual would have eligibility confirmed (medical history, temperature) before completing the DSF bioassay.

### Direct skin feeding procedure and standardisation

**Fig. 1:**
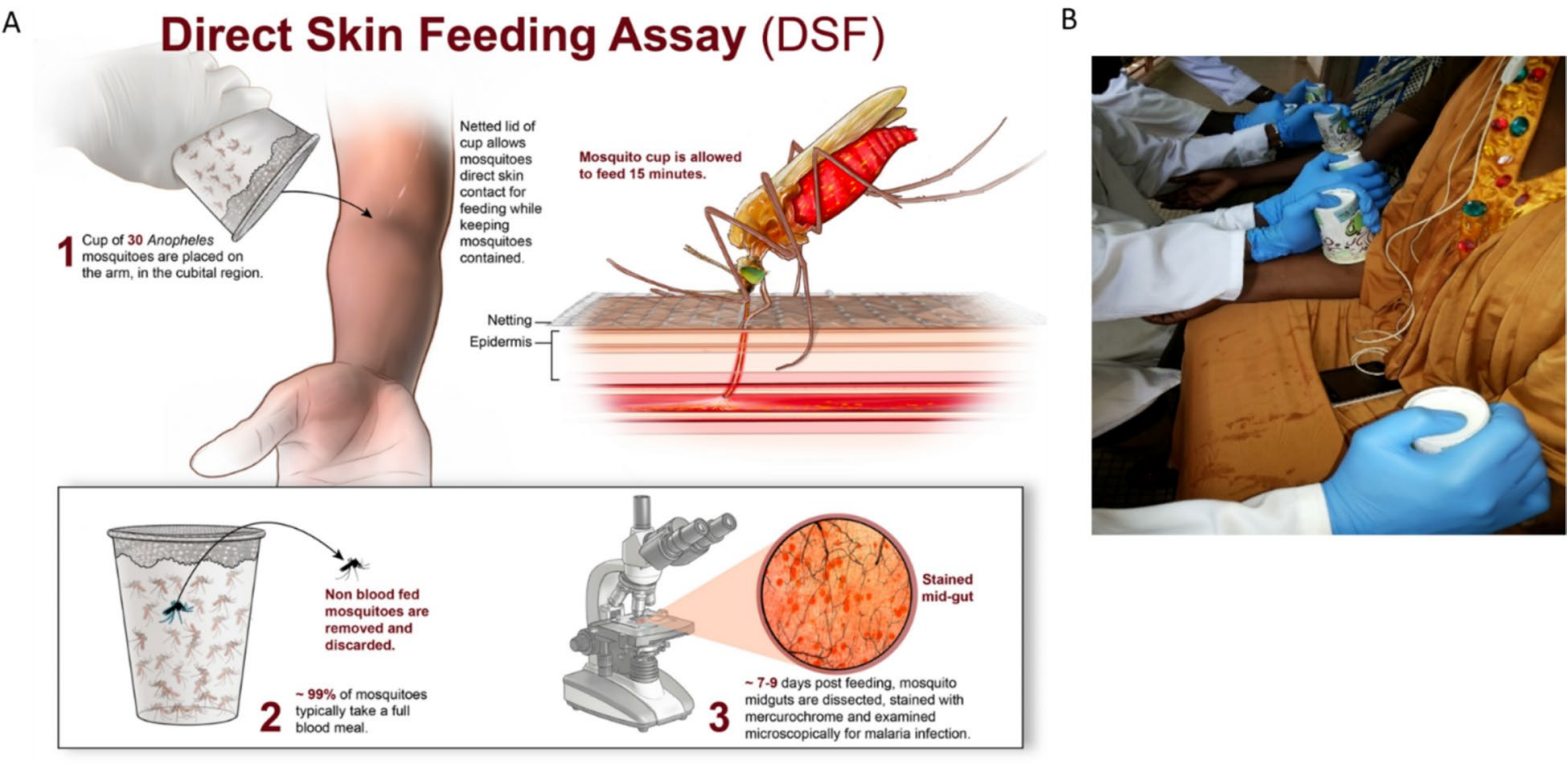
DSF Procedure. **A** Schematic of the DSF process; **B** photograph of DSF bioassay being conducted in the field by the MRTC study team. Illustration in panel A was generated by Malcolm Houston, Anita Mora, and Ryan Kissinger from Rocky Mountain Laboratories, National Institutes of Health, Hamilton, MT, USA

Direct skin feeding was conducted using two labelled cups of 30 mosquitoes placed directly on the proximal forearms, or if requested, the calves of volunteers for 15 min (Fig. 1A). Cups were labelled with the participant study identification number (ID), date of assay, and cup identifier (i.e. left or right cup). Volunteers were seated comfortably in a chair with their arms resting over their knees. A study team member sat on a low stool in front of each individual and held one cup firmly in each hand on the proximal forearm, anchoring the cup into the crook of the elbow (Fig. 1B). During the DSF, study volunteers were encouraged to listen to music or chat with the study team member to help distract them from any discomfort experienced during mosquito probing. Assay-specific parameters (e.g. time of assay, cup location, feed duration, age range of mosquitoes, number of mosquitoes, any issues with feed, number of mosquitoes blood fed) were recorded by the team on CRFs.

### Standardised volunteer follow up post-DSF

Following feeding, topical anti-pruritic cream was applied liberally and massaged into the forearms of all volunteers. Participants were solicited 24 h later to describe any adverse events related to the DSF bioassay procedure and be assessed by a trained study team member. Any individual who was identified as having a related AE the day after DSF was followed by the study team until the AE resolved, and the AE was documented on study-specific CRFs. Individuals who had a severe local or systemic response were excluded from future DSF bioassays and individuals were asked for this history before any DSF was performed as part of the eligibility criteria. Individuals were also free to opt out of DSF bioassays at any point for any reason.

### Analysis of infection and quality control

*Plasmodium* infection of mosquitoes was determined via midgut dissection and microscopic examination for the presence of oocysts. This was routinely completed seven days post-feeding. Mosquitoes were knocked down by vigorously shaking the cup, stunning the mosquitoes and enabling transfer to a petri dish on ice. Individual mosquitoes were then dissected under a stereo optical dissecting microscope (1-2X) in 0.5% mercurochrome. Intact midguts were removed and mounted on glass slides (two midguts per slide), left to stain for 20 min, then examined under light microscopy for the presence of oocysts. 10X magnification was used to scan for infection with oocysts, which were then counted using 20X or 40X magnification. If desired, individual midguts may have been removed from the slide after microscopic examination by lifting the cover slip and storing the stained midgut in appropriate buffer for molecular analyses.

To ensure accurate tracking of each DSF cup through the dissection and reading stages, petri dishes containing knocked down mosquitoes were labelled with the ID number and cup identifier (left or right cup), and one team member dissected both cups from a single feed. Each slide for reading contained two midguts mounted under separate cover slips, and the slide was labelled with ID number, cup identifier and mosquito number (i.e. first two mosquitoes dissected are 1 and 2, etc.). Loaded slides were placed onto a horizontal slide rack, and once all mosquitoes from both cups from a single individual were dissected and stained, they were handed over to a specialist reader. The reader noted the result from each individual midgut onto a source document tracking sheet, which was subsequently transcribed onto a results CRF with data entry verified by a second individual. All positive midguts were read and verified by a second reader.

### Midgut reading and quality control

In Mali, staff conducting entomology assays were divided into four main teams: mosquito production team, dissection team, midgut reading team, and a field team for performing the assays. Specialisation within the team allowed for a stepwise training and certification programme (whereby only the most experienced team members were cross-trained in all activities) and was optimal to support large-scale activities. When launching DSF for the first time or operating at a small scale, programmes may not need to define teams for different activities and instead manage with a single team whose members are trained on all activities.

The most rigorous and lengthy training was for individuals who were certified as midgut readers. These individuals underwent extensive training alongside a certified trainer to correctly identify oocysts of different stages (immature, mature, ruptured, folded/imperfect) and screen out common artifacts. Photos and pre-selected oocyst images were used initially before moving on to read freshly mounted and stained midguts. Before reading slides independently, trainees read slides alongside the trainer, tracking positive and negative slides, and counting oocyst numbers. All slides were double-read by a trainer, and once the trainee could read slides at a minimum of 1 positive midgut per minute and correctly identify a minimum of 95% of slides read, they were certified to work independently in a supervised environment. Newly certified readers continued to be closely supervised and were subject to spot-checking of reads until individuals had several months of experience, whereby correlation between trainer and trainee was expected to be 100% and readers were considered to be fully independent. Certification typically took around 1–3 months to complete and lasted for a two-year period, after which a certified individual was required to validate their credentials and renew certification by reading a set of 10 slides alongside a trainer. Typically, individuals who trained as readers have been with the entomology team for several years and gained at least six months training and experience in rearing mosquitoes, conducting DSF and dissecting midguts prior to training as readers.

Along with a rigorous training programmes for personnel and verification of infection by a second independent reader, validation of infected midguts was also achieved via post hoc molecular analyses for parasite speciation using PCR primers annealing to conserved *Plasmodium* spp. 18S reference sequences [7, 8]. This analysis confirms whether *Plasmodium* parasites were detected (yes/no) and the species of *Plasmodium*. A representative number of negative midguts (~ 5–10% of the total identified as positive) were also stored for later confirmation. Lastly and where possible, positive midguts were photographed using a camera attached to a light microscope to allow retrospective confirmation of findings, and the stored images provided a library for training and testing purposes.

## Results

### DSF acceptance and safety

The DSF bioassay was well-accepted by local Mali communities within the study sites. Acceptability of the DSF bioassay was assessed during three large clinical vaccine trials (NCT02334462, NCT02942277, NCT03917654), where missed visits or study procedures are monitored as potential protocol deviations and therefore documentation is rigorous. Among 772 vaccine trial participants, outright refusal to participate in any DSF bioassays was very low (6 individuals, 0.8%). Amongst the 766 individuals who agreed to participate in DSF bioassays, 14 (1.8%) refused DSF on one or more occasions (median 1 occasion, range 1–3). DSF were completed in 19,725/19,745 (99.9%) assays where the subject was deemed eligible.

Refusal and dropout rates were similar between studies with or without DSF bioassays. Importantly, the few participants who opted out of DSF in vaccine trials were kept on study and completed other study procedures, thus no specific withdrawals related to DSF bioassay were recorded.

**Fig. 2:**
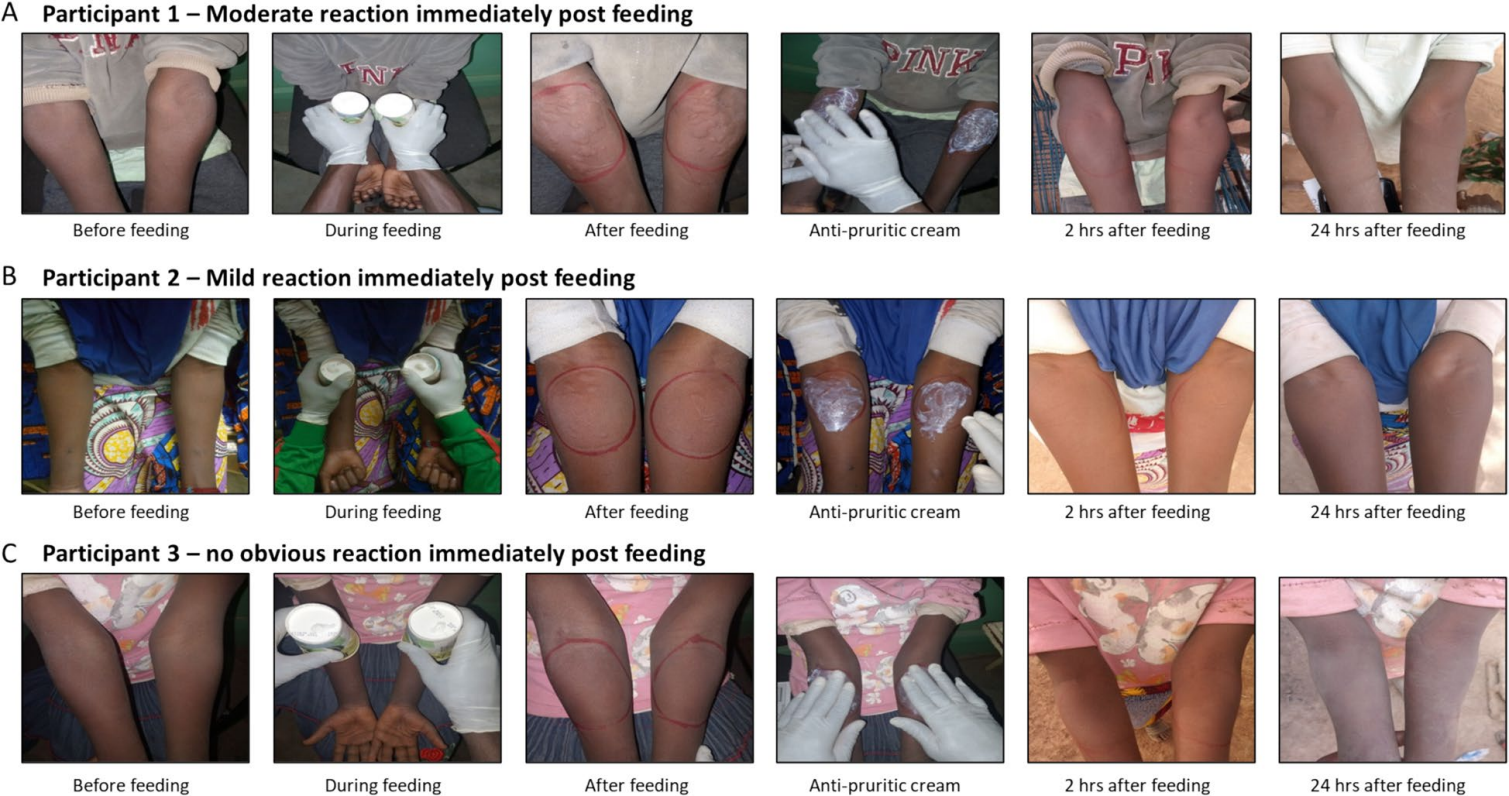
Expected skin reactions develop immediately after DSF procedures. **A** Participant 1 demonstrated a typical moderate reaction immediately post-feeding, which resolved by two hours after the feeding procedure. **B** Participant 2 demonstrated a mild reaction immediately post-feeding which resolved by two hours after the feeding procedure. **C** Participant 3 demonstrated no obvious reaction immediately post-feeding or in the following 24-h period

**Fig. 3:**
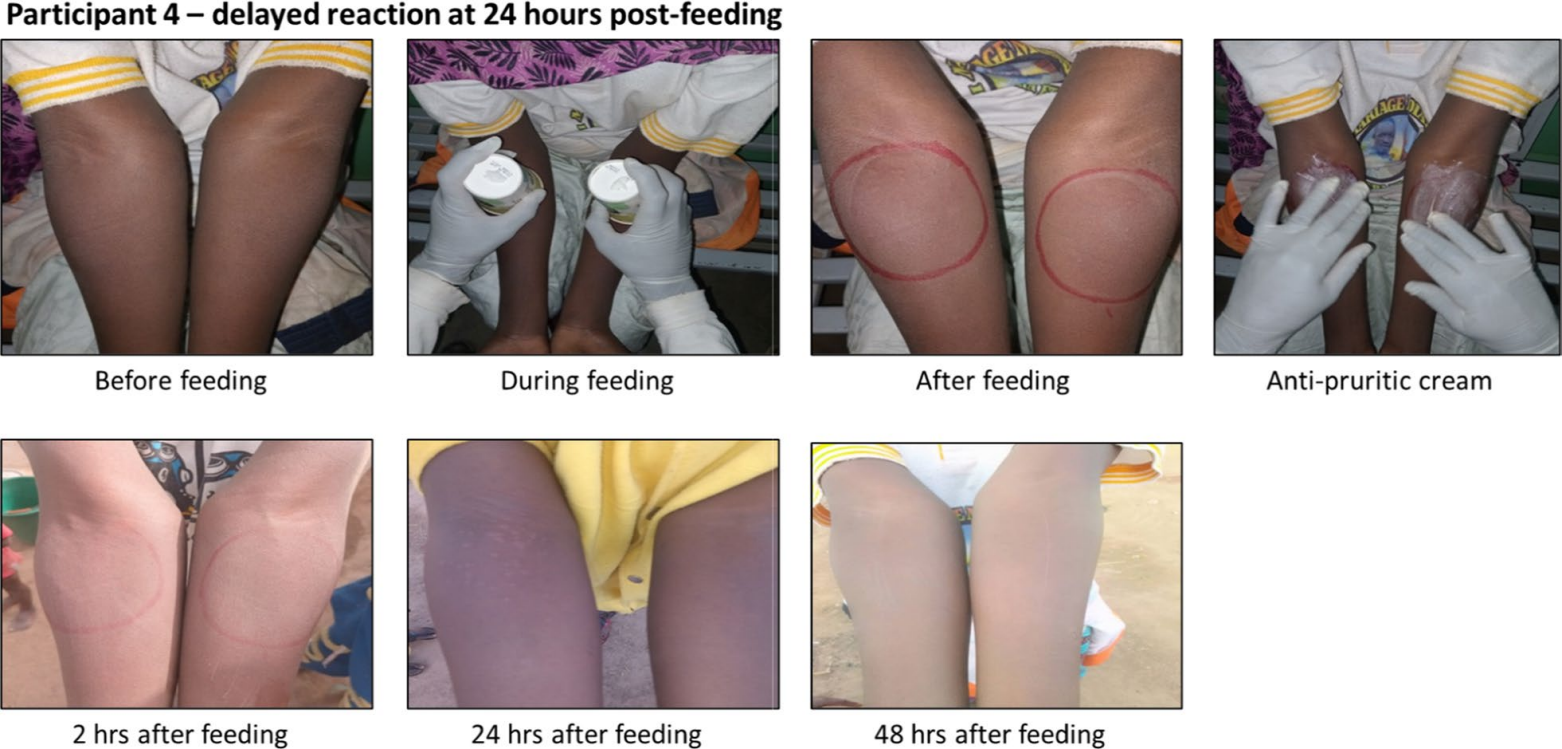
A delayed reaction rarely develops at 24 h post feeding. In contrast to the three participants in Fig. 2, study participant 4 demonstrated no reaction either immediately or at two hours post-feeding. However, by 24 h post-feeding, a slight skin reaction had developed which resolved by 48 h post-feeding. This individual was considered to have a mild Grade 1 pruritis AE per the grading criteria (Table 4)

In total, the study team conducted 37,984 feeding assays over a 15-year period and the assay was found to be safe and exceptionally well-tolerated, with only 12 adverse events (AEs), in 11 unique individuals, that met grading criteria and were related to the DSF procedure (0.032%; Table 3). All 12 AEs were recorded as Grade 1 or 2 (Table 4) local reactions that occurred at the site of mosquito feeding and resolved within 48 h of feed conduct. Responses to the DSF bioassay varied amongst individuals; common skin reactions immediately after feeding are illustrated in Fig. 2, and an example of a delayed reaction at 24 h post-feeding is shown in Fig. 3.

**Table 3:** Adverse event incidence in DSF across years

Year	Total No. DSF performed	AE incidence rate (per DSF)	Proportion of participants experiencing an AE	Age range of study participants	No. related AEs	AE grade	Symptom
2011	67	0	0/54	5–50 years	0		
2012	36	0	0/31	5–50 years	0		
2013	125	0	0/73	5–50 years	0		
2014	1023	0	0/135	5–50 years	0		
2015	2064	0.048	1/226	5–50 years	1	Grade 1	Pruritis
2016	1843	0	0/158	18–50 years	0		
2017	4866	0.10	4/217	18–50 years	5	All Grade 1	All Pruritis
2018	8843	0.023	2/833	5–65 years	2	Both Grade 2	Both Erythema
2019	10,685	0	0/1559	5–65 years	0		
2020	4536	0.022	1/731	5–65 years	1	Grade 2	Urticaria
2021	176	0	0/171	9–18 years	0		
2023	147	0.68	1/106	5–18 years	1	Grade 1	Erythema
2024	3007	0.067	2/469	5–18 years	2	Grade 2	Pruritis and Erythema
2025	566	0	0/375	5–18 years	0		
Total	37,984	0.032	11/5138		12

**Table 4:** DSF adverse event grading criteria

Post DSF local reaction AEs grading	Mild grade 1	Moderate grade 2	Severe grade 3	Potentially life threatening grade 4
Pruritus at site	Persist 24 h post DSF but does not require a repeat use of medication and does not interfere with activity	Require a repeated use of medication > 24 h or interferes with activity	Prevents daily activity	Emergency Room (ER) visit or hospitalisation
Erythema/redness	Persist 24 h post DSF and exceed the exposition surface with a diameter between 2.5–5 cm	Exceed the exposition surface and had a diameter between 5.1–10 cm	Exceed the exposition surface and had a diameter > 10 cm	Necrosis or exfoliative dermatitis
Pain	Persist 24 h post DSF but does not require a repeat use of non-narcotic pain reliever and does not interfere with activity	Requires a repeated use of non-narcotic pain reliever > 24 h or interferes with activity	Require any use of narcotic pain reliever or prevents daily activity	Emergency room (ER) visit or hospitalisation
Induration/swelling	Exceed the exposition surface and had a diameter between 2.5–5 cm but does not interfere with activity	Persist 24 h post DSF and had a diameter between 5.1 -10 cm or interferes with activity	Persist 24 h post DSF and had a diameter > 10 cm or prevents daily activity	Necrosis

### DSF bioassay performance metrics

**Fig. 4:**
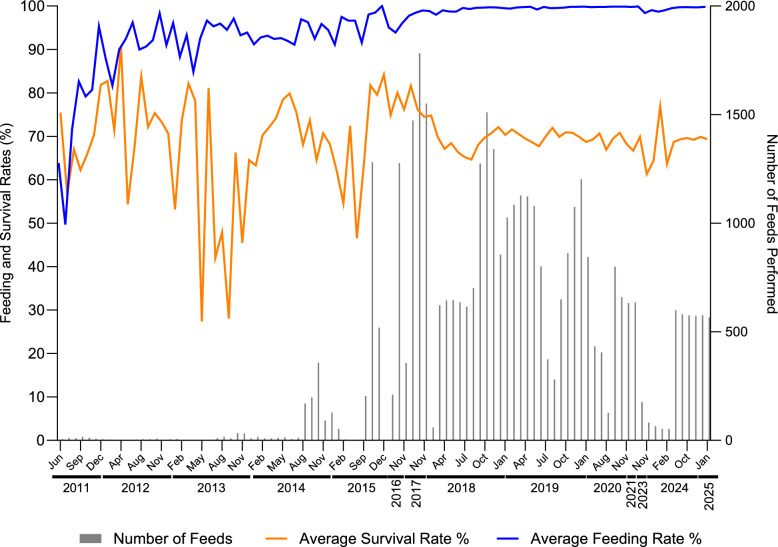
DSF bioassay performance metrics between 2011 and 2025. Graph presenting number of DSF bioassays conducted by month from 2011 to 2025 with corresponding average feeding rate (blue line) and average survival rate until time of dissection (day 7 post-feeding; orange line)

DSF bioassay utilises standardised measures including mosquito age at feeding, starvation time, anatomical feeding location and feeding time, all selected following optimisation studies conducted by the team [9]. Based on these standard practices, the average feeding and survival rates over time were recorded as performance metrics across studies from 2011–2025 (Fig. 4). In initial studies in 2011, mosquito feeding rates were as low as 50% but quickly improved as more assays were performed and since 2018 has been consistently over 99%. Similarly, post-feeding mosquito survival rate until time of dissection (day 7 post feeding) fluctuates slightly year on year depending on season and number of feeds conducted but survival rates are consistently high across studies (Fig. 4). The average feeding rate over the course of the studies presented here was 98.8%, and average survival rate to midgut dissection was 71.2% (Table 5).

**Table 5:** Scale up of DSF in Mali over time

Year	No. DSF performed	No. DSF performed < 18 yo	No. DSF Performed ≥ 18 yo	No. Mosq Fed	No. Mosq dissected	Average feeding rate % (SD)	Average survival rate % (SD)
2011	67	38	29	2535	1703	74.0 (22.5)	66.2 (19.8)
2012	36	14	22	1958	1463	91.5 (6.7)	74.6 (11.5)
2013	125	5	120	7115	3787	95.0 (4.5)	53.2 (27.7)
2014	1023	26	997	57,981	38,827	94.5 (6.7)	68.5 (17.1)
2015	2064	3	2061	120,516	90,165	97.3 (4.6)	78.8 (14.6)
2016*	1843	0	1843	52,741	37,022	94.4 (5.3)	78.7 (15.5)
2017	4866	0	4866	287,356	210,669	98.4 (2.1)	77.3 (9.9)
2018	8843	2287	6556	526,994	343,485	99.3 (1.4)	68.6 (7.1)
2019	10,685	7894	2791	638,790	425,075	99.6 (1.0)	70.1 (6.6)
2020	4536	4201	335	271,700	178,028	99.8 (0.7)	69.0 (6.7)
2021	176	160	16	10,549	7000	99.9 (1.3)	69.9 (8.3)
2023	147	147	0	8704	5451	98.7 (2.4)	62.6 (7.6)
2024	3007	2988	19	179,783	124,717	99.6 (1.4)	69.4 (6.3)
2025	566	565	1	33,895	23,501	99.8 (0.9)	69.3 (5.4)
Total	37,984	18,328	19,656	2,200,617	1,490,893	98.8 (3.3)	71.2 (10.4)

*15 mosquitoes/cup were used in the 2016 study vs. a standard of 30 mosquitoes/cup in other studies

Rates of positive DSF (i.e., at least one mosquito infected during the DSF) were typically low. The highest rates of DSF positivity were observed when individuals known to be carrying parasites or gametocytes were selected for DSF (Table 6), which yielded an average DSF positivity rate across studies of 18.4%. DSF positivity varied across years which can be attributed partly to natural yearly variations in parasite carriage but also to the age of study participants, gametocyte positivity rate and time of year DSF bioassays were conducted. The highest rate of DSF positivity (50%) occurred in 2012 but this is not unexpected, as 85% of DSF assays were performed on individuals carrying gametocytes by microscopy.

**Table 6:** DSF infectivity to mosquitoes over time from individuals without recent malaria vaccine, selected to undergo DSF based on blood smear or rapid diagnostic test results

Year	Total No. DSF performed	No. positive DSF (%)	No. infected mosquitoes (%)	Parasite status at DSF (%)^3^ asexual parasites only; gametocytes ± asexual; RDT +	Mean oocyst count (range) in infected mosquitoes only
2011^1^	43	9 (20.9%)	58 (5%)	58.1%; 41.9%; n/a	2.6 (1–20)
2012^1^	34	17 (50%)	301 (21.9%)	14.7%; 85.3%; n/a	7.7 (1–47)
2013^1^	70	8 (11.4%)	75 (3.6%)	50.0%; 50%; n/a	6.9 (1–29)
2014^1^	75	17 (22.7%)	82 (2.8%)	37.3%; 62.7%; n/a	4.5 (1–80)
2015^1^	6	0	0	33.3%; 66.7%; n/a	–
2023^2^	67	6 (9%)	49 (2%)	34.3%; 9.0%; 100%	22.3 (1–99*)
2024^2^	25	2 (8%)	18 (1.7%)	56.0%; 28.0%; 100%	1.8 (1–4)
Total	320	59 (18.4%)	583 (5.1%)		7.7 (1–99*)

^1^Denotes trials where individuals were selected for feeds based on blood smear parasite positivity (no routine RDT performed)

^2^Denotes trials where individuals were selected for feeds based on a positive RDT (blood smear read retrospectively and did not affect DSF bioassay selection)

^3^Parasite status was determined in different studies either by blood smear positivity or RDT positivity. Blood smear status was split into 1) individuals who had only asexual parasites on blood smear (number with asexual parasites/number of individuals) and 2) individuals who had gametocytes on blood film irrespective of accompanying asexual parasites on blood smear (number with gametocytes /number of individuals). RDT + individuals are those that returned a positive RDT test (number with + RDT test/number of individuals)

^*^Denotes that highest allowable count is 99 oocysts; those higher than 99 are recorded as 99 + but counted as 99 for all analyses

n/a: Not applicable; RDT not performed

In 2014, the DSF bioassay inclusion criteria during vaccine trials was updated to include all participants irrespective of blood smear status. Logistically, this change in process streamlined clinic visits so the DSF bioassay could be performed immediately after a subject’s clinical evaluation, without waiting for blood smears to be stained and examined. Scientifically, this change was warranted by the observation that participants with negative blood smears may nevertheless transmit during DSF on infrequent occasions. As expected, the switch to feeding on all study participants was accompanied by a drop in DSF positivity rate, with an average of 1.6% across all studies (Table 7). While blood smear status is a key metric influencing DSF outcome, so too is seasonality and age of study participants; data presented in Table 7 comprise many feeds conducted in the dry season and many feeds conducted on adults who, as a group, yield fewer transmission events compared to school-age children.

**Table 7:** DSF infectivity to mosquitoes over time in individuals without recent malaria vaccine, undergoing regularly scheduled DSF bioassay regardless of parasitaemia status

Year	Total N DSF Performed	N Infected DSF (%)	N Infected Mosquitoes (%)	Mean Oocyst Count (Range), Infected Mosquitoes only
2014	413	12 (2.9%)	139 (0.9%)	7.7 (1–46)
2015	534	18 (3.4%)	145 (0.6%)	2.9 (1–13)
2016	464	4 (0.9%)	10 (0.7%)	2.4 (1–7)
2017	2491	21 (0.8%)	115 (0.1%)	3.4 (1–26)
2018	6308	83 (1.3%)	574 (0.2%)	9.3 (1–99*)
2019	9482	136 (1.5%)	1116 (0.3%)	12.9 (1–99*)
2020	2798	104 (3.7%)	734 (0.7%)	7.2 (1–98)
2021	79	0	0	–
2024	2902	40 (1.4%)	393 (0.3%)	6.3 (1–40)
2025	566	6 (1.1%)	59 (0.3%)	35.7 (1–99*)
Total	26,037	424 (1.6%)	3285 (0.3%)	9.6 (1–99*)

^*^Denotes that highest allowable count is 99 oocysts; those higher than 99 are recorded as 99 + but counted as 99 for all analyses

### Data interpretation at the level of participant, mosquito, or oocyst

**Fig. 5:**
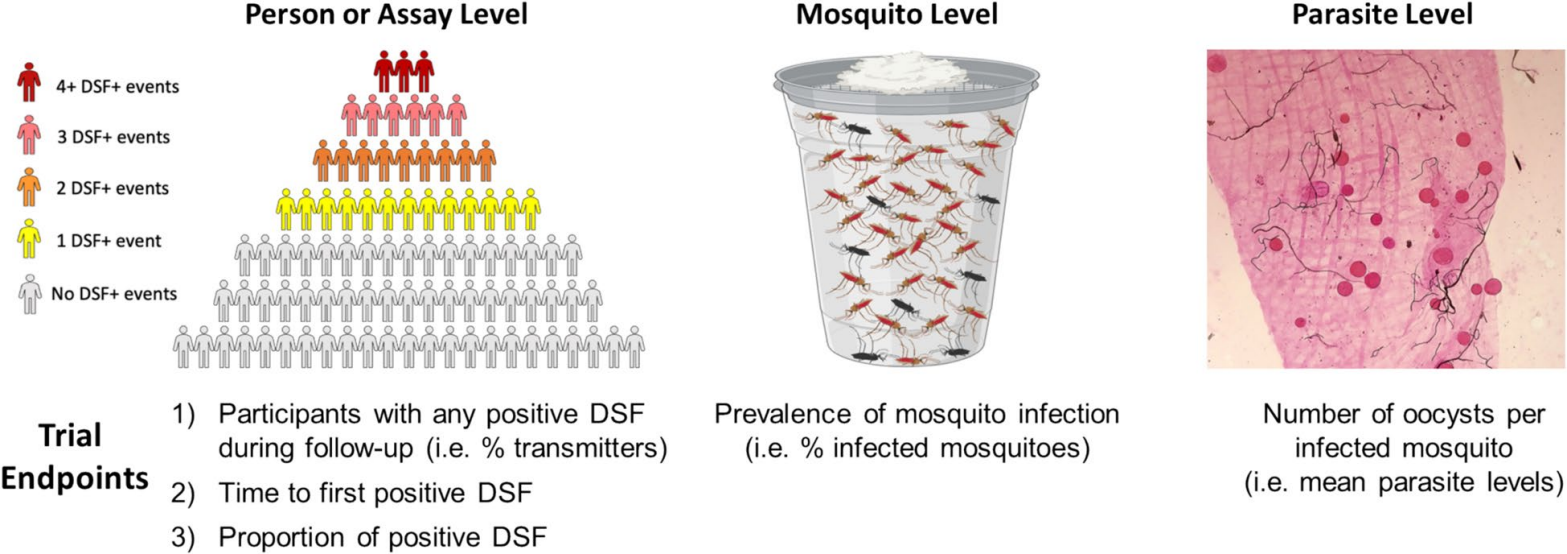
Data interpretation and endpoints for analysis. Pictograph depicting various levels to interpret DSF data: 1. Person or assay level – proportion of transmitters (i.e., individuals with at least 1 positive DSF during follow up) or proportion of positive assays; 2. Mosquito level – proportion of infected mosquitoes; 3. Parasite level – number of oocysts per mosquito in positive assays. The image of the mosquito level panel was created in BioRender. Gorres, J. (2025) https://BioRender.com/m1f1f9a

DSF data can be interpreted according to different endpoints depending on the study objectives (Fig. 5). The number of participants with one or more positive DSFs can be analysed either as the proportion of “transmitters” in the population or as the time to first transmission, and the number of positive DSFs over time as percentage of all DSF or as the incidence rate of transmission (person or assay level). Individual midgut data can be analysed as the proportion of infected mosquitoes (mosquito level). The number of oocysts in each infected mosquito can be analysed as a measure of intensity of transmission (parasite level). To date, multiple TBV trials have utilised these approaches to analyse and interpret DSF data [7, 8]; figures from these publications present infected mosquito counts per DSF subject, showing the zero-inflated pattern and heterogeneity of mosquito infections between and within individuals over time, and an additional example is provided in Supplementary Figure S1.

## Discussion

TBVs prevent the spread of malaria by targeting mosquito sexual stage parasites, thereby reducing the number of new malaria infections within a community [3, 26]. As the vaccine does not directly prevent infection of the vaccinee, measurement of TBV clinical efficacy remains complicated, requiring utilisation of surrogate measurements of efficacy as an alternative to large cluster-randomised trials. For decades, the SMFA has been the gold standard assay for assessing malaria TBV activity. However, as field trials of TBV have progressed [7, 8], SMFA has not been predictive of vaccine efficacy measured by DSF surrogate endpoints [7]. SMFA has other disadvantages compared to DSF (Table 7), with limited throughput and the requirements for specialised equipment as well as in vitro preparation of mature *P. falciparum* gametocytes. SMFA is also limited by its reliance on laboratory strains of *P. falciparum*, high inter-assay variability in transmission intensity, and perhaps most importantly its failure to mimic aspects of naturally occurring parasite transmission.

In contrast to SMFA, both DMFA and DSF can be performed in a field clinic and measure transmission of naturally circulating parasites (﻿Table﻿ 8). In three studies with paired DSF and DMFA data, DSF detected a significantly greater number of positive feeds than DMFA (P < 0.0001) (unpublished findings); similar results have been described by others [10, 20, 27–29]. In addition to lower infection rates compared to DSF, DMFA is usually only performed at a relatively small scale (< 50 assays per day) as they are a resource intensive procedure [30] requiring blood draws and strict temperature control before and during mosquito feeds.

DSF performed with mosquitoes or other arthropods has long been used as a xenodiagnostic tool for numerous vector-borne diseases. DSF bioassay that measures malaria parasite transmission requires fewer steps and resources than alternative procedures. DSF can be completed indoors or outdoors at the field clinic, and with appropriate staffing has been scaled to ~ 120 assays per day. Throughput can be increased further with expansion of insectary rearing facilities and increased staffing. All mosquito assays require a reliable insectary with proper containment procedures and personnel skilled in mosquito dissection and reading, but DSF is advantageous in its simplicity, requiring no expensive specialised equipment or even electricity at the feeding site (see Table 8).

**Table 8:** DSF as a tool to measure TBV efficacy compared to DMFA and SMFA

	DSF	DMFA	SMFA
Mimics natural transmission	Yes	PARTLY	No
Safety Concerns	No	No	No
Easily scalable	Yes	No	No
Sufficient throughput for vaccine efficacy analyses	Yes	Maybe	No
Requires laboratory equipment to perform			
Insectary	Yes	Yes	Yes
Parasite culture facility	No	No	Yes
Laboratory equipment e.g. centrifuge and water bath	No	Yes	Yes
Cost-effective at scale	Yes	No	No
Conducted at field site in real time	Yes	Yes	No
Requires additional blood draw	No	Yes	Yes

As the largest body of data collected on the DSF bioassay to date, these studies confirm DSF to be safe and well-tolerated, with exceptionally low rates of solicited AEs attributed to the assay in a population with frequent exposure to the mosquito species used in the assay. Immediate and short-term skin reactions occurred (Fig. 2), but in nearly all cases resolved by 24 h post-DSF and therefore did not meet criteria to be defined as an AE. All events that met AE criteria were local in nature, did not exceed Grade 2, and resolved within 48 h of the feeding procedure (Tables 3–4). The liberal use of topical antipruritic cream immediately post-feeding likely minimised skin reactions and their severity. In future studies, new AE solicitation will be extended to 48 h post-feeding to enable capture of rare or delayed reactions. Current studies conduct DSF every 2 weeks and no AE related to DSF persisted to the next scheduled DSF bioassay.

Previous studies examined allergic reactions to mosquito bites and documented both local reactions (e.g. Skeeter’s syndrome) and systemic reactions (such as generalised urticaria and anaphylaxis) [31, 32] but these events were not observed in over 37,000 assays that were conducted, suggesting such reactions occur very rarely in this study population. The low reactogenicity to mosquito bites may reflect the intense lifelong exposure of this population to the mosquito species used for DSF where entomological inoculation rates as determined by human landing catch are as high as 137 to 167 infectious bites per person over one transmission season [23]. In controlled feeding studies on volunteers, mosquito bite reactogenicity increased with repeated initial exposures to a new vector but thereafter decreased with continued exposure and apparent desensitisation [32–34].

DSF bioassay was performed according to protocols approved by Malian IRB and was well-accepted by the participating Malian communities. Incorporation of DSF as a standard procedure in research protocols did not delay study approvals nor did it have a negative impact on study enrolment or dropout rates observed in either adults or children. Successful implementation of the DSF bioassay was facilitated by early engagement with community leaders followed by community information meetings regarding upcoming studies. This ensured studies and assays including DSF were well-explained and allowed concerns or questions to be addressed proactively.

While these data draw exclusively from study sites in Mali, ongoing collaborations include multiple partner sites across Africa where DSF has been or is being introduced for longitudinal cohort studies of parasite transmission [35, 36]. Potential risks and confounding factors may differ between study areas. For example, *Anopheles*-borne lymphatic filariasis has been documented by the National Programme for Filariasis throughout west Africa. However, this was not considered a potential risk to these studies for the following reasons: filariasis is nearing eradication in Mali, the *An. coluzzii* species used for DSF does not contribute to vertical transmission of filariasis, and the colony originated from collections in 2008.

To date, the DSF bioassay has been approved by all ethical bodies and accepted by local communities at these diverse sites. To facilitate implementation at new sites, an informational packet to explain DSF bioassay purpose, safety record, monitoring, and standard operating procedures has been prepared and shared with study teams.

## Conclusions

The DSF bioassay is a scalable and safe method for evaluating TBV efficacy, offering several advantages including close mimicry of naturally occurring transmission, simplicity of performance and standardisation, sufficient throughput to support late-stage vaccine trials, and biological plausibility to measure surrogate efficacy endpoints for trials of TBV. While the proportion of positive DSF bioassays overall is low, sufficient transmission endpoints are generated for statistical analysis of vaccine effects, supporting utilisation of the DSF bioassay as a surrogate efficacy endpoint for TBV trials.

## Supplementary Information

Additional file 1.

## Data Availability

All data generated or analysed during this study are included in this published article. The datasets used and/or analysed during the current study are available from the corresponding author on reasonable request.

## Abbreviations

AE: Adverse event
CRF: Case report form
DMFA: Direct membrane feeding assay
DSF: Direct skin feeding assay
EIR: Entomological inoculation rate
FMPOS: Faculty of Medicine-Odonto-Stomatology
ID: Participant study identification number
LMIV: Laboratory of Malaria Immunology and Vaccinology
MRTC: Malaria Research and Training Center (Bamako, Mali)
NIAID: National Institute of Allergy and Infectious Diseases
NIH: National Institutes of Health
PBMS: Participant biometric matching system
PCR: Polymerase chain reaction
SMFA: Standard membrane feeding assay
SOP: Standard operating procedure
TBV: Transmission-blocking vaccine
