# Supplementary material for: Mosquito direct skin feeding bioassay: 15 years of experience and a standardised approach in Mali
Source: Malar J. 2026 Feb 4;25:134. doi: 10.1186/s12936-025-05726-7 (PMC12964775; doi:10.1186/s12936-025-05726-7)

Supplementary Figure S1. Inter-individual and temporal heterogeneity of parasite transmission from Malian adults to mosquitoes by DSF bioassay over two malaria seasons. Each subject who received comparator vaccine in a trial of Pfs230D1-EPA/AS01 in Malian adults [7] is depicted as a continuous timeline over two seasons; study termination within a season is indicated by a truncated line within that season; subject dropout/termination between season is indicated by an absent line in the second season. Small black dots indicate negative DSFs, small red dots within red circles indicate positive DSFs, and the size of red circles represents percentage of infected mosquitoes (scale shown in the inset key).

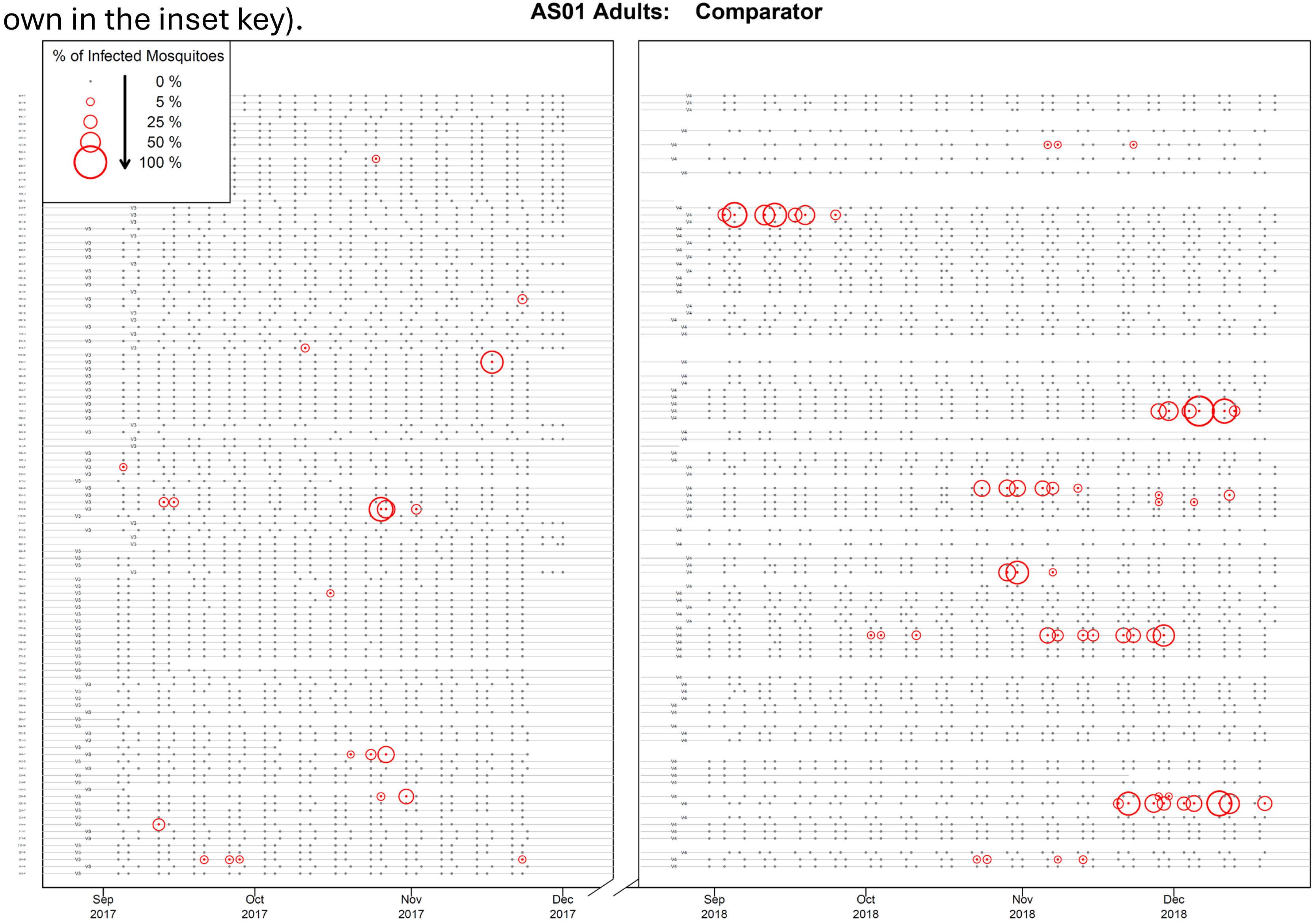

Supplement: Supplementary file 1 — Additional file 1. [file 12936_2025_5726_MOESM1_ESM.pdf]
